# Accessibility and availability of maternal and reproductive health care services: ensuring health equity among rural women in Southern India

**DOI:** 10.1186/s12875-024-02369-6

**Published:** 2024-04-29

**Authors:** Geetha Jeganathan, Sampath Kumar Srinivasan, Senthilkumar Ramasamy, Pitchaimani Govindharaj

**Affiliations:** 1 Gramalaya NGO, Tiruchirappalli, Tamil Nadu India; 2 Department of Sociology & Population Studies, Bharathiar University, Coimbatore, Tamil Nadu India; 3 Health System Strengthening, State Health Resource Centre, Raipur, Chhattisgarh 492001 India; 4 Department of Allied Health Sciences, Sri Ramachandra Institute of Higher Education and Research (DU), Chennai, Tamil Nadu India

**Keywords:** Accessibility, Availability, Maternal health, Reproductive health, Rural women

## Abstract

**Introduction:**

The health of women is of particular concern because they often have greater health needs than men and many women still lack access to quality healthcare services, preventing their ability to attain the best possible level of health. Hence, this study aimed to assess the accessibility and availability of health services among rural women.

**Methods:**

A household survey was conducted by using a multi-stage sample with 407 married women aged 18–45 years, having at least one child and living in Tiruchirappalli District, Tamil Nadu were recruited for this study. A semi-structured questionnaire was used to collect data about the demographic status, and accessibility and availability of health services.

**Results:**

Of the 407 respondents, 70% were aged between 26 and 40 years, 73% were working as farmers and labourers and 77% were living in nuclear families. 71% of them had enjoyed hospital facilities near their residence and 83% of the hospitals were run by the government. In village health nurse service (VHN), 34% of the respondents had received all services from VHN and 86% did not face any kind of gender inequality and almost all of them were satisfied with the service provided by the VHN. Almost all the respondents (98%) were satisfied with the availability of health services and 92% of them benefited from the government scheme related to childbirth.

**Conclusion:**

This study showed that overall, the women were satisfied with the availability of healthcare services and reproductive health services. Moreover, almost all of them benefited from the government scheme related to childbirth.

**Supplementary Information:**

The online version contains supplementary material available at 10.1186/s12875-024-02369-6.

## Introduction

United Nations (UN) has recognized universal access to reproductive healthcare as a global health priority, and Universal Health Coverage (UHC) aims to ensure that everyone receives essential health services, encompassing prevention, promotion, treatment, rehabilitation, and palliation [1]. Health is considered a fundamental human right, involving not only the delivery of timely and appropriate healthcare but also ensuring that everyone can access the needed health services without financial strain, anytime and anywhere [2].

The health of women is of particular concern because they often have greater health needs than men and many women still lack access to quality healthcare services, preventing their ability to attain the best possible level of health. Reproductive and maternal health care remains a major challenge to the public health system [3]. In India, approximately 1.3 million women have died due to maternal causes in the last two decades. In 2020, almost 800 women died every day, equating to one woman per every two minutes, from preventable causes related to pregnancy and childbirth. Approximately 95% of maternal deaths occur in low and lower-middle-income countries due to non-utilization or under-utilization of maternal and reproductive services [4].

Health equity can be achieved when everyone can attain the full potential for health and well-being by removing obstacles to optimal health [5]. Ensuring the availability and accessibility of comprehensive, high-quality healthcare services is important for preventing and managing diseases, as well as promoting health for all individuals. In India, 70% of the population resides in rural areas. Women living in rural parts are considered a vulnerable group in terms of maternal and reproductive health due to various factors such as lack of support, knowledge, poor connectivity, and transportation facilities [6]. Any inequality or unavailability of basic reproductive healthcare services can lead to a higher risk of morbidity and mortality in both the mother and children [3].

Empowering women about their rights and health status will help them to seek appropriate health services. The Sustainable Development Goals (SDGs) ensure access to sexual and reproductive health-care services by 2030. Recently, India has made a significant improvement in maternal and reproductive health care [6]. However, it is important to sustain this progress and study periodically to know the accessibility and availability of health services in developing countries like India, especially among rural women. Hence, this study aimed to assess the accessibility and availability of maternal and reproductive health services among rural women in Tamil Nadu, India.

## Materials and methods

### Study setting

A community-based cross-sectional descriptive study was undertaken among women residing in the rural villages of the Tiruchirappalli district. The study was carried out between November to December 2017.

Tiruchirappalli is one of the oldest districts present in the centre of Tamil Nadu, it has a diverse population with various socio-economic and cultural backgrounds. The district is surrounded by rural agricultural villages with 50.87% population living in this area. A similar pattern of diversified and rural population was observed in the state of Tamil Nadu (51.60%) in southern India. This sample will be representative of rural women facing similar challenges in the state of Tamil Nadu.

### Participant eligibility

The study included all adult married women aged between 18 and 45 years, living with their husbands, having at least one child, capable of independent communication, and willing to participate. A written informed consent was obtained from the respondents after explaining the purpose of the study and assuring confidentiality.

### Semi-structured questionnaire

A semi-structured questionnaire was prepared to collect the demographics and the accessibility and availability of maternal and reproductive health services. The accessibility and availability of health services which include the availability of drugs, ambulance services, diagnostic equipment, child care, village health nurse services, and reproductive health services, including sterilization, contraceptive materials, and government schemes related to childbirth [Additional file S1].

### Sample size and sampling techniques

According to 2011 Census, the total population of Tiruchirappalli district living in rural areas is 1,384,257, with 688,552 males and 695,705 females with a sex ratio of 1,010 females for every 1,000 males and average literacy was 76.69%. The average literacy rate in India and the female literacy rate are 74.04% and 65.46% respectively. The average literacy of Tamil Nadu and the female literacy are 80.09% and 73.44%. The literacy of females in Tamil Nadu is higher than the average among Indian women but the literacy average in rural areas is similar to Tamil Nadu. Tamil Nadu can be a place as a general rural area in India. In administration, the district has three Revenue Divisions, nine Taluks and 14 Community Development Blocks. There are 471 Revenue Villages out of which, 431 villages are inhabited in this District [7].

The Researcher adopted a multistage sampling technique, to determine the sample frame. For the study, five blocks were selected from a total of 14 blocks of Tiruchirappalli district by using the lottery method and subsequently chosen one village panchayat from each of the five Blocks, again by adopting the lottery method. All the identified hamlets from the five-village panchayat were selected for the study and the number of hamlets varied from two to seventeen in each of village panchayat. The total target population of all the hamlets, who fulfilled the sampling criteria for the study population, was 2,036 women. The minimum sample required at a 95% confidence interval was calculated to be 385, with 5% additional data collected. According to the adjusted sample size, approximately 407 rural women were included in this study from the study population through simple random sampling.

### Data collection

A first author was assigned to recruit respondents, describe the study to the respondents, obtain informed consent and perform the face-to-face interview with the assistance of trained and qualified female field investigators. The interview schedule consists of information about the present demographic and accessibility and availability of health services. All interviews were conducted in the vernacular language, Tamil. The interview was conducted in strict privacy after building rapport with the respondents. On average, each interview lasted about 30–40 min, including the time spent on the establishment of rapport.

### Data analysis

The data were entered into a Microsoft Excel database and analysed using SPSS (Statistical Package for Social Sciences) version 26.0 for analysis. Simple frequencies, percentages and distribution measures were calculated for demographics and accessibility and availability of health services.

## Results

Of the 407 respondents, age ranged between 21 years to 45 years and 48% of the women were aged between 31 and 40 years. Three hundred and seventy-eight (79%) of them were married at the age of 18 & above years of their age. All the respondents had attained formal education and among them, 42% and 43% had attained education up to secondary level and higher secondary & above respectively. Hundred and eighty-one (45%) of them were working as laborer and 29% were doing agriculture work. Three hundred and thirty-two (78%) of the respondents were living in a nuclear family and 46% of their family were earning between Rs. 5,000 to Rs. 10,000 (**Showed in** Table 1). A majority of Indian households’ income is between 125,000 and 500,000 Indian rupees a year in the financial year of 2021 [8].

**Table 1:** Demographic profile of the respondents (*n* = 407)

Variables	Frequency (n)	Percentage (%)
Age of the respondents (mean ± SD in years)	34.47 ± 6.51	
21–25 years	39	9.6
26–30 years	90	22.1
31–35 years	99	24.3
36–40 years	98	24.1
41–45 years	81	19.9
Age at Marriage (mean ± SD in years)	20.79 ± 2.89	
Below 18 years	86	21.1
19–25 years	292	71.7
Above 25 years	29	7.1
Education		
Primary	64	15.7
Secondary	173	42.5
Higher Secondary & above	170	41.8
Occupation		
Labour	181	44.5
Agriculture	117	28.7
Govt & Private, Business	38	9.3
Housewife	71	17.4
Type of family		
Nuclear family	316	77.6
Joint family	91	22.4
Family income per month (mean ± SD in Indian Rupees)	8626.29 ± 7478.68	
Up to Rs.5,000	143	35.1
Rs.5,000–10,000	189	46.4
Above Rs.10,000	75	18.5

### Accessibility of health services

Table 2 shows the accessibility status of respondents to the nearest health care facility for maternal and reproductive health care services. The nearest health care facility mentioned by the majority 337 (83%) of respondents were public health hospitals [216 (53.1%) government hospitals, 121 (28.8%) primary health centres]. Of the 216 respondents who selected the government hospital as the nearest healthcare facility, it was accessible to 119 respondents and not accessible to 97 respondents. Similarly, 121 respondents had chosen the primary health centre as the nearest, it was accessible to 116 respondents and not accessible to 5 respondents. Among 70 respondents who selected the private hospital as nearest, reported that it was accessible to 55 respondents and not accessible to 15 respondents (**Showed in** Table 2).

**Table 2:** Accessibility of health care service by the respondents (*n* = 407)

Status	Maternal and Reproductive Health care Services					Total (*n* = 407)	
	Accessible *n* = 290			Not Accessible (*n* = 117)			
	F	%		F	%	F	%
Government hospital (GH)	119	41.0		97	82.9	216	53.1
Primary Health Centre (PHC)	116	40.0		5	4.3	121	29.8
Private hospital	55	19.0		15	12.8	70	17.2
Total	290	100.0		117	100.0	407	100.0

Of the 407 respondents, 92% used the bus as a mode of transport for hospital visits and 78% of them could reach the hospital within 30–45 min. Two hundred and fifteen (53%) of them had to wait up to 30 min to consult a doctor and 98% were satisfied with the health services provided by the health institution and 55% of them had spent up to Rs.50 for every visit (**Showed in** Table 3).

**Table 3:** Mode of transport and time took to reach hospital by the respondents (*n* = 407)

Variables	Frequency (n)	Percent (%)
Availability of Hospital nearby from the Place of Residence		
Available	290	71.3
Not available	117	28.7
Mode of Transport		
Bus	375	92.1
Two-Wheeler	21	5.2
By walk	5	1.2
Car	6	1.5
Time took to reach hospital (mean ± SD in minutes)	43.40 ± 14.72	
30 min	120	29.5
45 min	196	48.2
60 min	89	21.9
More than 60 min	2	0.5
Waiting time for doctor consultation (mean ± SD in minutes)	43.23 ± 21.11	
30 min	215	52.8
60 min	186	45.7
more than 1 h	6	1.5
Satisfaction of service		
Satisfied	399	98.0
Not satisfied	8	2.0
Expense per visit (mean ± SD in Indian Rupees)	180.37 ± 274.96	
Up to Rs.50	224	55.0
Rs.50–100	67	16.5
Rs.100–200	40	9.8
Rs.200–500	45	11.1
Above Rs.500	31	7.6

In village health nurse service (VHN), 34% of the respondents had received all services from VHN and 50% of them had received only the service of vaccine. The majority of them (86%) did not face any kind of gender inequality and almost all of them were satisfied with the service provided by the Village Health Nurses (VHN) (**Showed in** Table 4).

**Table 4:** Services of village health nurse (*n* = 407)

Status	Frequency (n)	Percent (%)
Services of Village Health Nurse		
All	140	34.4
Maternal care	2	0.5
Dispensing Medicine	62	15.2
Vaccination	203	49.9
Gender Inequity		
Faced	57	14.0
Not faced	350	86.0
Satisfaction of Nursing Services		
Satisfied	406	99.8
Not satisfied	1	0.2

Table 5 shows the health services availability of 407 respondents. Almost all the respondents were satisfied with the availability of health services.

**Table 5:** Health services availability (*n* = 407)

Status	Frequency (n)	Percent (%)
Drugs		
Adequate	405	99.5
Inadequate	2	0.5
Equipment’s		
Available	327	80.3
Not available	80	19.7
Child care		
Available	399	98.0
Not available	8	2.0
Emergency treatment		
Available	387	95.1
Not available	20	4.9
Ambulance		
Available	400	98.3
Not available	7	1.7
Referral system		
Available	382	93.9
Not available	25	6.1
Difficulties in getting appointment		
Difficult	26	6.4
Easy	381	93.6
Availability of staff		
Inadequate	48	11.8
Adequate	359	88.2

In the availability of reproductive health services, 98% of them reported that the availability of sterilization consultation and the health provider being equipped to conduct sterilization and to offer contraceptive material. 99% of the respondents reported that the hospitals provided delivery facilities service, where they obtained health services and 92% of them benefited from the government scheme related to childbirth and they were satisfied (**Showed in** Table 6).

**Table 6:** Reproductive health service availability of the respondents (*n* = 407)

Status	Frequency (n)	Percent (%)
Sterilization consultation		
Available	397	97.5
Not available	10	2.5
Facilities to perform sterilization		
Available	400	98.3
Not available	7	1.7
Contraceptive material		
Available	398	97.8
Not available	9	2.2
Facilities for child birth		
Available	403	99.0
Not available	4	1.0
Satisfaction of Government scheme		
Satisfied	374	91.9
Not satisfied	33	8.1

## Discussion

The health and well-being of the people of a country largely depend on well-developed, accessible and effective healthcare infrastructure. India is the second most populous country in the world, and it also has the largest rural population in the world. This study reported on the status of accessibility and availability of maternal and reproductive health services among rural women.

In this study, more than two-thirds of respondents reported that hospitals were accessible near their residences and the majority were public health hospitals. Nearly all the respondents used public buses as the mode of transport to reach the hospital, and three-fourths of them reached the hospital within 30–45 min. About half of them waited up to 30 min to avail doctor’s consultation and the rest of them were waiting up to 60 min. Almost everyone was satisfied with the health services provided by the health institution and more than half of them had spent up to Rs.50 for every visit.

The Government of India launched the National Rural Health Mission (NRHM) in 2005 to strengthen primary health care, the Accredited Social Health Activist (ASHA) program was started to address the problem of safe motherhood [9]. The ASHA workers act as interfaces between the community and the public health system, aiming to improve access to health services, raise community awareness about health and its social determinants, and support the primary healthcare system in facilitating care, specifically for maternal and child health [9]. In this study, almost all respondents received health services related to maternal and reproductive healthcare, with one-third of them receiving a comprehensive range of services from Village Health Nurses, and all respondents expressed satisfaction with the services provided by the village health nurses.

In terms of availability of health services, almost all the health service centres had stocked enough drugs, enough medical equipment to treat patients, with provision for child care service, emergency treatment facilities and ambulance service for emergency care. Almost all the health service centre reported referral system, with tertiary care centre except few health services (6%) which did not have any the referral system.

Regarding difficulties in availing health care by the respondents, majority of them did not experience any denial of services from the health centre and also majority of them did not experience any difficulties in getting appointment.

Studies from India, found that socio-economic factors and service delivery environment as important determinants influencing maternal health services [10, 11]. A study from Uttar Pradesh, India, observed that 83% of the women had utilized antenatal care services [12]. In this study, the availability of reproductive health services, almost all the women were satisfied with the availability of reproductive health services; sterilization consultation, equipment to conduct sterilization, contraceptive material and maternity facilities service. Incidentally, all the respondents benefitted by the Government program, related to pregnancy and child birth.

## Conclusion

The periodic awareness campaigns play an important role in promoting the optimal utilization of public health services among the general population. It is necessary to conduct targeted awareness programs for the women residing in rural remote areas, which focus mainly on maternal and reproductive health care services.

Regular long-term follow-up is necessary to monitor women’s health, especially for reproductive-related health. The school awareness programme is an effective and efficient method to transmit knowledge, related to reproductive health, for adolescent students. This method of programme creates awareness about reproductive health to the students and helps to spread awareness to the people.

### Electronic supplementary material

Below is the link to the electronic supplementary material.

Supplementary Material 1

## Data Availability

The dataset used or analyzed during the current study will be available from the corresponding author upon reasonable request.

## Abbreviations

VHN: Village Health Nurse
UN: United Nations
UHC: United Health Coverage
SDGs: Sustainable Development Goals
SPSS: Statistical Package for Social Sciences
GH: Government Hospital
PHC: Primary Health Centre
NRHM: National Rural Health Mission
ASHA: Accredited Social Health Activist
