# Supplementary material for: Accessibility and availability of maternal and reproductive health care services: ensuring health equity among rural women in Southern India
Source: BMC Prim Care. 2024 Apr 29;25:145. doi: 10.1186/s12875-024-02369-6 (PMC11059574; doi:10.1186/s12875-024-02369-6)
Supplement: Supplementary file 1 — Supplementary Material 1 [file 12875_2024_2369_MOESM1_ESM.doc]

**Annexure -1**

**Interview Schedule**

**District:**

**Block:**

**Village: STUDY NO.**

**----------------------------------------------------------------------------------------------------------**

**I- Demographic Profile**

1. Age :______________________________________
2. Educational status : ______________________________________
3. Years of schooling : ______________________________________
4. Occupation : ______________________________________
5. Family income : ______________________________________
6. Type of family : ______________________________________
7. Age at marriage : _____________________________________

**II- Health services**

**II.1. Accessibility**

1. Do you have hospital nearby your home? Yes / No

If yes, type of hospital______________________________________________

If no, distance / time to reach the hospital ______________________________

1. What is the mode of transport to reach hospital? ____________________________
2. Is there outpatient facility in the hospital? Yes / No

If yes, service timings ______________________________________________

If no, why _______________________________________________________

1. How much you are spending average cost per visit to go to hospital? ___________
2. How long you have to wait to meet the Doctor? ____________________________
3. Do you have satisfaction with the services provided by hospital? Yes / No

If yes, please specify ______________________________________________

If no, why? ______________________________________________________

1. Do you have regular visit by VHN / ASHA to your village? Yes / No
2. What type of services are providing by VHN / ASHA? ______________________
3. Do you get the receipts for your payment? Yes / No
4. Do you satisfy with the services provided by VHN / ASHA? Yes / No
5. Do you feel any gender inequalities in providing treatment? Yes / No

**II.2. Availability**

1. Is there enough drugs / medical equipments in the hospital? Yes / No
2. Is there on-time referral done to other hospitals / Doctors? Yes / No

If no, why? ______________________________________________________

If yes, which referral venue suggest by the doctors? ______________________

Mention distance and time __________________________________________

1. Do you feel any exclusion of services from the hospital? Yes / No

If yes, specify ____________________________________________________

1. Do you face inadequate workforce (doctor, nurse, unskilled labour)? Yes/No If yes, specify ____________________________________________________
2. Do you have any difficulty in getting appointment to meet Doctor? Yes / No
3. Is the hospital has emergency treatment facilities? Yes / No

If it is not there, where are you going for emergency? _______________________

1. Is the hospital has child care facilities? Yes / No

If it is not there, where are you going for child care? _____________________

1. Is there ambulance services at the hospital for emergency? Yes / No

If no, why _______________________________________________________

1. Is there family planning consultation separately in the hospital? Yes / No
2. Is there family planning facilities in the hospital? Yes / No
3. Is there enough contraceptive materials available? Yes / No
4. Is there delivery facilities / equipments at the hospital? Yes / No
5. Are you satisfied with Govt. schemes relates to maternal health? Yes / No

If yes, please specify _______________________________________________

If no, what is the reason? ____________________________________________
